# Military nursing in tactical pre-hospital care: structure, action, and challenges in high-risk contexts

**DOI:** 10.1590/0034-7167-2025-0183

**Published:** 2026-01-26

**Authors:** Fernanda Idamares da Silva Souza, Priscilla Valladares Broca, Fábio José de Almeida Guilherme, Débora Fernanda Haberland, Thiago de Souza Louro, Eric Rosa Pereira, Thiago Augusto Soares Monteiro da Silva, Alexandre Barbosa de Oliveira

**Affiliations:** I Universidade Federal do Rio de Janeiro. Rio de Janeiro, Rio de Janeiro, Brazil; II Força Aérea Brazileira. Rio de Janeiro, Rio de Janeiro, Brazil; III Universidade Federal do Estado do Rio de Janeiro. Rio de Janeiro, Rio de Janeiro, Brazil; IV Exército Brazileiro. Manaus, Amazonas, Brazil; V Faculdade Souza Marques. Rio de Janeiro, Rio de Janeiro, Brazil

**Keywords:** Military Nursing, Nurse’s Role, Disasters, Emergency Medical Services, Warfare and Armed Conflicts., Enfermería Militar, Rol de la Enfermera, Servicios Médicos de Urgencia, Desastres, Guerra y Conflictos Armados.

## Abstract

**Objectives:**

to understand, in light of Anthony Giddens’ Structuring Theory, how military nursing professionals act in tactical pre-hospital care, highlighting training, management, and care practices in risk contexts, and structural challenges that put pressure on their practice.

**Methods:**

a qualitative, descriptive study with 15 participants recruited through chain sampling. Data collection took place between March 2023 and January 2024 using semi-structured interviews. The corpus was processed in Interface de R pour les Analyses Multidimensionnelles de Textes et de Questionnaires and analyzed using Descending Hierarchical Classification.

**Results:**

four classes emerged, organized into three axes: leadership in training; resource management in hostile environments; challenges related to communication and scarcity of supplies.

**Conclusions:**

military nursing demonstrates agency by adapting practices and producing innovation even in the face of rigid hierarchical structures. Tactical pre-hospital care must be institutionalized, investing in training, technologies, and scientific production that consolidate this field as strategic for operational health.

## RESUMEN

**Objetivos:**

comprender, a la luz de la Teoría de Estructuración de Anthony Giddens, cómo actúan los profesionales de enfermería militar en la atención táctica prehospitalaria, destacando las prácticas de formación, gestión y atención en contextos de riesgo y los desafíos estructurales que presionan su práctica.

**Métodos:**

estudio cualitativo y descriptivo con 15 participantes reclutados mediante muestreo en cadena. La recopilación de datos se realizó entre marzo de 2023 y enero de 2024 mediante entrevistas semiestructuradas. El corpus se procesó en la Interface de R pour les Analyses Multidimensionnelles de Textes et de Questionnaires y se analizó mediante clasificación jerárquica descendente.

**Resultados:**

surgieron cuatro clases, organizadas en tres ejes: liderazgo en el entrenamiento; gestión de recursos en entornos hostiles; desafíos relacionados con la comunicación y la escasez de suministros.

**Conclusiones:**

la enfermería militar demuestra capacidad de acción al adaptar prácticas y generar innovación incluso frente a estructuras jerárquicas rígidas. Es necesario institucionalizar la atención prehospitalaria táctica, invirtiendo en formación, tecnologías y producción científica que consoliden este campo como estratégico para la salud operacional.

## INTRODUCTION

The role of military nursing in war and armed conflict scenarios has been historically documented, but there is a lack of scientific literature that systematically examines nursing performance in tactical pre-hospital care (TPHC), particularly in Brazil. The available literature focuses largely on historical accounts or conceptual analyses, highlighting a scarcity of empirical studies investigating the operational, training, and managerial challenges faced by these professionals in high-risk contexts^(1-3)^.

TPHC thus emerged as a response to the demands imposed by war and armed conflict, configuring itself as an emergency healthcare model focused on high-risk situations. To this end, protocols adapted to different tactical realities-civilian and military-were developed to improve the quality of care provided and improve immediate field response. A prime example of this systematization is Tactical Combat Casualty Care (TCCC), established in 1996 by the Naval Special Warfare Command and the United States Special Operations Command, with the central objective of reducing combat casualties, ensuring adequate care for the wounded, and contributing to the success of operational missions^(4)^.

TCCC is based on three core principles: Care Under Fire; Tactical Field Care; and Casualty Evacuation Care. These principles enable military healthcare professionals, whether nurses or not, to act effectively, even under enemy fire, providing emergency basic and advanced life support care during the care and safe evacuation of casualties in the tactical field^(4)^.

Professionals involved in TPHC are classified into three levels of practice, as specified by the Department of Defense Health Agency (DHA) Joint Trauma System (JTS): Level I - all military service members; Level II - non-medical military personnel in combat operations; and Level III - military medical professionals, paramedics, nurses, and soldiers. This classification is widely adopted internationally, being adapted by armed forces and law enforcement agencies in various parts of the world^(5-7)^. In this context, nursing has established itself as a central profession in training promotion and development. In Brazil, nurses and nursing technicians have been acting as instructors and educational agents for TPHC^(8)^.

When the COVID-19 pandemic hit the U.S., military healthcare professionals, including nurses, physicians, and technicians, were mobilized to reinforce civilian hospital staff. In 2020, a task force of 140 military healthcare personnel facilitated the distribution of safe and effective vaccines, diagnostics, and therapies in record time, directly contributing to the preservation of more than a million lives^(3)^. Such evidence reinforces the strategic relevance of military nursing in strengthening operational health, both within the armed forces and in the interface with civilian public health.

In light of Anthony Giddens’s Structuration Theory^(9)^, it is understood that military nursing practice in TPHC develops in a field tensioned by the duality between structure and agency. On the one hand, institutional norms, hierarchical codes, and operational protocols form a set of rules and resources that limit and regulate professional practice, but on the other, military nurses exercise agency (protagonism) by reinterpreting, negotiating, and resignifying these structures in their daily work. Thus, TPHC in the military field should not be understood merely as a locus for the technical application of pre-established norms, but as a dynamic and relational social space in which practices are continually (re)constructed in interaction with structural conditions. This perspective allows us to recognize that the challenges faced by military nursing are not limited to technical or logistical barriers, but relate to broader structuring processes, with direct implications for consolidating evidence-based practices and strengthening military nursing as a field of knowledge and specialized practice^(1,8)^.

Given this scenario, it is essential to analyze how structural and operational constraints interfere with military nursing professionals’ practice in TPHC. It is also important to understand how these constraints impact the production of knowledge, the quality of care provided, and the institutionalization of military nursing as a strategic dimension of operational health.

## OBJECTIVES

To understand, in light of Anthony Giddens’ Structuring Theory, how military nursing professionals work in TPHC, highlighting training, management, and care practices in risk contexts, and the structural challenges that put pressure on their practice.

## METHODS

### Ethical aspects

The research protocol was submitted to and approved by the Research Ethics Committee of the proposing institution. All participants signed the Informed Consent Form. To ensure anonymity, interviewees were identified by alphanumeric codes, with the prefix “NP” for nursing professionals, followed by a sequential number corresponding to the order of interviews.

### Study design

This descriptive, exploratory and qualitative research study was developed according to the methodological guidelines recommended by the COnsolidated criteria for REporting Qualitative research.

### Methodological procedures

Data collection was carried out between March 2023 and January 2024. Participant recruitment was carried out using the snowball sampling technique, widely recognized in qualitative research as an effective strategy for accessing specific or hard-to-find groups, especially in restricted institutional contexts such as the military^(10)^. The first informant-a nurse in the Brazilian Armed Forces-was identified during an event hosted by an academic nursing league in emergency care at the *Universidade Federal do Rio de Janeiro*, where the lead researcher served as president of the student organization. Following this initial contact, the remaining participants were progressively recommended by the interviewees themselves. This networking process enabled access to professionals with expertise in TPHC, fostering a diversity of experiences and profiles until theoretical data saturation was reached.

### Study setting

The interviews were conducted individually and remotely via Google Meet^®^. This virtual data collection method offered greater flexibility to participants, considering that many of them were on active duty or deployed in different regions of the country. In addition to geographic barriers, the strategy also enabled overcoming logistical barriers.

### Sample composition and selection criteria

The sample for this study consisted of 15 military professionals, including nurses and nursing technicians, affiliated with the Armed Forces (Navy, Army, or Air Force) and Auxiliary Forces, such as the Military Police and State Fire Departments. Initially, 16 participants were recruited; however, one of them was excluded from the interview stage due to assignment to an operational mission during the collection period, resulting in a total of 15 active participants in data production.

Professionals with institutional ties to the Armed Forces or public security forces, technical or higher education in nursing, and at least one year of direct experience in TPHC were included in the study to ensure the participation of individuals with experience in the operational field under investigation. Professionals who were away from their duties at the time of data collection, whether due to leave or reserve duty (paid or unpaid), were excluded, thus ensuring the timeliness and relevance of the reports collected.

### Data collection and organization

Data collection was conducted through semi-structured interviews organized along two central axes. The first axis focused on participant sociodemographic and professional characteristics, including variables such as sex, age group, length of nursing training (technical and/or higher education), and job tenure in TPHC. The second axis included open-ended questions aimed at understanding professional experience in TPHC context, including topics such as military nursing responsibilities, challenges faced in the operational field, training and continuing education processes, logistical barriers, and perceptions of interprofessional communication in tactical environments.

The interviews were audio-recorded. They were then transcribed verbatim and submitted for validation by the interviewees themselves to ensure the reliability and credibility of the information collected. The average length of the interviews was 150 minutes, with a range of 60 to 150 minutes.

Data collection was concluded based on the theoretical saturation criterion, i.e., when the interviews began to present thematic recurrence and an absence of new relevant data, as recommended in the literature on the qualitative approach^(11,12)^.

### Data analysis and interpretation

Data analysis was conducted using Descending Hierarchical Classification (DHC), using the *Interface de R pour les Analyses Multidimensionnelles de Textes et de Questionnaires* version 0.7 alpha 2 software. This software is widely recognized for its robustness in analyzing textual data and extracting lexical patterns in qualitative research.

The analytical process involved the following stages: 1) text *corpus* pre-processing, with standardization of spelling, elimination of linking words without semantic load, and material preparation for segmentation; 2) *corpus* segmentation into Initial Context Units and Elementary Context Units, allowing detailed analysis of the frequencies and relationships between the most significant terms; 3) DHC processing, which organized the data into a dendrogram of lexical classes, identifying recurring linguistic and semantic patterns in participants’ discourses; and 4) class interpretation, considering the contexts and relationships established among participants’ discourses, in light of Giddens’ Structuration Theory, which supported the discussion of the findings.

Class interpretation was guided by the foundations of Anthony Giddens’ Structuration Theory^(9)^, with an emphasis on the concepts as follows: “structure”, understood as the set of institutionalized norms, rules, and resources that organize and condition action; “agency”, as individuals’ ability to act reflexively, resist, adapt, or transform these structures; and “routine”, understood as the way in which practices are reproduced or modified in daily life. This approach allowed us to identify the relational dynamics between the structural dimensions and military nursing professionals’ actions in TPHC.

## RESULTS

### Sociodemographic and professional profile

The sample consisted of 15 military nursing professionals, of which 13 (86.7%) were male and two (13.3%) were female. Concerning professional training, 53% of participants had a degree in nursing and 47% were nursing technicians, evidencing a diverse composition regarding the level of education and responsibilities in the healthcare field.

Analysis of age range revealed an experienced professional group, with 100% of respondents over 36 years of age. Training time ranged from 5 to 20 years for both technical and higher-level professionals. Specific experience in TPHC, considering service in the Armed Forces and Auxiliary Forces, ranged from 5 to 15 years, reinforcing participants’ expertise and practical maturity in this study.

### Text *corpus* lexical analysis

*Corpus* lexical analysis revealed 715 text segments, of which 586 were used, accounting for 81.96% of the analyzed material. The total number of occurrences was 25,105 words, forms, or vocabulary, of which 2,300 were distinct words and 2,001 words had a single occurrence (hapax).

**Figure 1:**
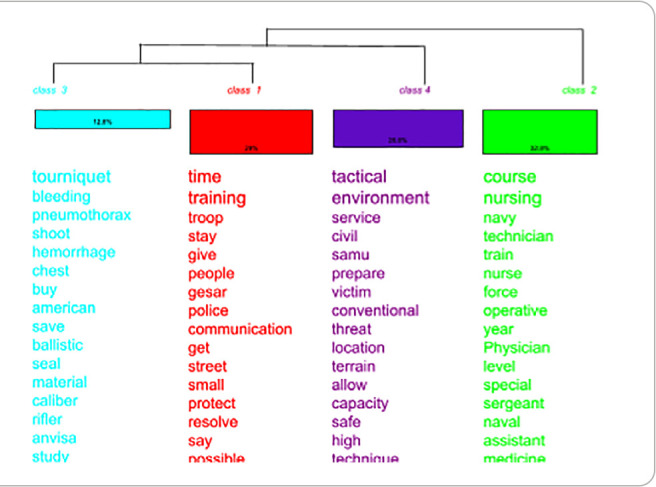
Descending Hierarchical Classification dendrogram, Rio de Janeiro, Rio de Janeiro, Brazil

*Source: IRaMuTeQ® 0.7 alpha 2 software.*

DHC allowed the *corpus* to be organized into four distinct classes, resulting in the division of discourses into two main branches. The first branch consisted exclusively of class 2, while the second branch included classes 1, 3, and 4. The resulting dendrogram highlights the discourse segmentation and semantic cohesion of the analyzed content (Figure 1).

Semantic and categorical analysis identified three major central topics, organized around four lexical classes. This framework enabled a deeper understanding of the military nursing work dynamics in TPHC, highlighting the challenges, strategic functions, and operational barriers faced in the tactical context.

### Emerging class interpretation

*Corpus* lexical analysis revealed four lexical classes, organized into three major central topics that express different dimensions of military nursing practice in TPHC. This perspective allows us to understand that military nursing practices are not limited to the mechanical application of pre-established protocols. On the contrary, they involve continuous processes of social production and reproduction, in which operational routines are reflexively recreated, combining tacit knowledge, technical improvisation, and situational adaptation.

Each of the identified classes, accompanied by the corresponding text segments, derived directly from participants’ statements, is presented below, with the purpose of illustrating the empirical dimensions that underlie the emerging topics.

### Reproducing and transforming practice: military nursing professionals as training agents (class 2)

This category highlights the structuring role of military nursing professionals as training agents, capable of reproducing and transforming institutional practices within TPHC. Training in TPHC occurs at multiple levels and settings, permeated by hierarchies, traditions, and training gaps that require nursing professionals to play a continuous role in team development, especially at levels 2 and 3. In this process, nurses and technicians not only reproduce institutional norms but also contribute to their redefinition by adapting content to operational realities, promoting methodological adaptations, and competing for spaces of technical and political legitimacy.

*Nurses play a key role in training, especially for military personnel at levels 2 and 3, who will be on the front lines.* [...] *in Brazil, few physicians and nurses work in this field. We need to ensure how this knowledge is transmitted, practiced, and consolidated at levels 2 and 3.* (NP1)

*Throughout my career, I’ve always found myself teaching constantly. When a nurse fresh from the officer’s course takes on the role, they often have little or no experience in the tactical context* [...]. *It’s up to me to start from scratch and train them. This applies to physicians, nursing technicians, everyone.* (NP10)

*Within the military, we see healthcare professionals as multipliers of tactical pre-hospital care. They are the ones who will be out in the far reaches of Brazil, training and retraining combatants.* (NP9)

*I fought hard to have nurses and physicians placed on the same level as physicians in advanced tactical prehospital care, and I succeeded. We’re there at the advanced level-physicians and nurses. If I hadn’t been there representing the profession, if a physician had gone, for instance, the legislation would be different.* (NP6)

### Management and structuring of actions in hostile environments: military nursing strategies in tactical pre-hospital care (class 4)

This category highlights the strategic role of military nursing as an organizing and coordinating agent for logistical and care actions within TPHC. In situations marked by resource scarcity, technical demands, and imminent risks, nursing professionals play a central role not only in direct care but also in managing supplies, coordinating teams, and planning tactical responses.

The following excerpts illustrate the main axes of this class:

*If we face a disaster, we need to calculate: how many tourniquets can be used? How many bandages will be needed? This type of discussion is essential to ensure assistance.* (NP9)

*When I train soldiers in tactical response, I’m also dealing with management issues. This expert advice makes the response more effective, especially in disasters. It’s necessary to plan what will be disseminated to the troops and what improvements to implement. I work with management, teaching, and logistics.* (NP9)

*Military work places nurses and technicians in extremely challenging situations, requiring clinical reasoning, leadership, and the application of knowledge in hostile and high-stress environments*. (NP11)

*Even public security institutions haven’t yet grasped that the ‘safe scene’ model is designed for countries with no history of war. In Brazil, especially in Rio de Janeiro, we live in a state of permanent urban conflict.* (NP1)

### Structural barriers and technological challenges in tactical prehospital care: communication and scarce resources (classes 1 and 3)

This category articulates two axes of analysis central to the reality of military nursing in TPHC: interprofessional communication (class 1) and the scarcity of material and technological resources (class 3).

As for class 1, communication in tactical contexts is strained by inflexible hierarchical structures, compromising coordination among members of the multidisciplinary team. Difficulty recognizing nurses’ technical authority and other professionals’ lack of knowledge of specific protocols generate disruption, delays, and even failures in care.

*Due to military doctrine, sometimes we try to discuss a procedure with the attending physician and mention the TC3 or another specific protocol, and this complicates things, as they don’t understand what we’re talking about. The situation is aggravated when we deal with officers who aren’t open to learning. I’ve had many problems arriving at the emergency room with an injured patient because of this.* (NP11)

*Hierarchy does get in the way. Often, those in leadership positions aren’t from the healthcare field and don’t understand certain technical recommendations. Before a crisis, there’s resistance; after the crisis hits, everyone falls silent, and you become the “owner of the truth”. I’ve lost patients because of this. It’s difficult because, even with prior guidance, they don’t always listen. Hierarchical authority prevails, and we have to comply, even when it’s not the best decision.* (NP15)

*Communication within the troop itself is often the main problem. In some operations, we manage to enter the community with the patrol, leave the ambulance outside, and continue with our backpacks and equipment. However, due to communication failures, strategic entry and evacuation points are not always effectively defined, which compromises the entire operation.* (NP2)

*Communication sometimes fails. We need to build trust to overcome these difficulties. These days, even with cell phones, there’s still noise that directly impacts service.* (NP6)

Class 3 addresses challenges faced by TPHC related to the scarcity of essential supplies, such as tourniquets, hemostatic agents, and specialized bandages, which poses significant barriers to care delivery. Even with established protocols, the lack of materials compromises their implementation and effectiveness.

*There’s no point in training and training if, at the time of the incident, there’s no quality equipment available. For instance, if someone is shot and we use a tourniquet, that item is disposable. It needs to be replaced later. And that’s expensive.* (NP7)

*Nowadays, if we want work equipment, we have to buy it. Many professionals have become aware of this: if you don’t buy it, you could die. That’s why it’s common to see personal tourniquets attached to uniforms.* (NP2)

*Many of the materials we use in tactical prehospital care arrive through international exchanges, especially with the United States. They donate them to us. However, when these items expire and there is no new exchange scheduled, we are forced to purchase them out of pocket.* (NP11)

## DISCUSSION

The results indicated a predominance of male professionals in TPHC, which reflects the institutional legacy of the Armed Forces, marked by an organizational culture that prioritizes physical strength, strict discipline, and tactical operationality^(13,14)^. All interviewees were over 36 years old, which indicates an experienced professional body with a consolidated trajectory in high-risk contexts.

It was also observed that the professionals interviewed actively seek to improve their preparation for action in complex scenarios, which involve armed conflicts, urban violence, and disasters^(15)^. This trend is also reported in studies conducted in conflict zones in Ukraine and in North Atlantic Treaty Organization missions, where military nurses combine logistical, training and clinical responsibilities, being recognized as leaders in the field^(16-18)^.

In light of Anthony Giddens’ Structuration Theory^(9)^, it is possible to interpret that classes express a dynamic process in which subjects, while being conditioned by the organizational and normative structures of the military field, also exercise agency, i.e., the capacity to act, adapt and transform these same structures.

Class 2, which highlighted the role of nursing professionals as training agents, reaffirms the assumptions of Giddens’ Structuration Theory^(9)^, as it reveals how these individuals reconfigure institutional norms based on their daily practice. By mediating tactical knowledge with care techniques, military nurses exercise “agency”, constructing solutions that are simultaneously operationally appropriate and pedagogically innovative^(4,8,15,19,20)^. This reality converges with military training models adopted by countries such as Israel and the United Kingdom, where nurses play a central role in tactical trauma education, including in civilian missions^(21,22)^.

In light of Giddens’ Structuring Theory^(9)^, the pedagogical practice carried out by these professionals expresses a dynamic process of agency, in which the transmission of knowledge is not limited to formal protocols, but also involves the incorporation of tacit knowledge, adapted to the demands of daily military life.

Class 4 demonstrated the ability of military nurses to perform managerial functions in environments characterized by scarcity and unpredictability. The practices reported, such as training planning, input management, and tactical response coordination, reflect these individuals’ ability to shape organizational structure based on practice^(8,21)^. This agency, in Giddens’s terms, becomes essential in unstable areas, as demonstrated by the recent experiences of the U.S. Army and the German Armed Forces in supporting the population during floods^(23,24)^.

In light of Giddens’ Structuration Theory^(9)^, this category reflects the dialectical relationship between structure and action: despite being embedded in a rigid normative system, military nurses exercise agency by negotiating, adapting, and transforming operational practices. This agency manifests itself in the way these professionals manage decision-making flows, optimize field logistics, and influence technical training and leadership processes in critical scenarios.

Barriers to interprofessional communication (class 1), in turn, reveal tensions between nursing’s technical authority and the hierarchical norms of the military structure. Interoperability between healthcare professionals and operational command, identified as a challenge, is equally present in other emergency response situations. The lack of mutual understanding between technical and operational areas compromises the fluidity of care, requiring interagency training and unified tactical communication protocols^(13,25,26)^.

Class 3 highlighted the scarcity of supplies as a critical barrier to the implementation of international TPHC protocols, such as TCCC. The shortage of tourniquets, hemostatic agents, and protective equipment directly impacts response capacity and team safety. In the U.S., recent legislation has prioritized funding for tactical trauma kits for civilian and military units, recognizing the role of tactical nurses as central providers of care^(2-4,8,22)^. In Brazil, however, the dependence on donations or personal acquisition by professionals highlights the absence of structural policies for operational health.

In light of Giddens’ Structuration Theory^(9)^, it is understood that, even when conditioned by rigid structures, such as military norms, institutional hierarchies and budgetary limitations , nursing professionals exercise agency when developing strategies to maintain care effectiveness in high-risk scenarios.

This evidence reinforces that, even in the face of structural constraints, military nursing professionals exercise creative agency, reinterpreting norms and adapting practices in defense of life. Giddens’ framework allows us to understand that the structure is not immutable, but constantly renegotiated by the individuals who inhabit it.

Given this scenario, it is urgent to incorporate successful international experiences, expand the institutionalization of TPHC as a strategic field, and invest in technical and managerial nursing training. Strengthening collaborative networks, specific regulatory regulations, and the production of robust scientific evidence are promising avenues for consolidating the role of military nursing in the contemporary context of complex crises.

### Study limitations

This study faced limitations related to the specific nature of the military field. Many military nursing professionals were frequently on missions or strategic deployments when contacted, which made it difficult to access a larger number of participants. This context, inherent to the institutional and cultural dynamics of military corporations, ultimately limited the possibility of expanding the diversity of voices heard throughout the research. For future studies, collaboration with institutional sectors could pave the way for a more structured approach to different operational units, expanding the scope and representativeness of the findings.

### Contributions to nursing and health

The results of this research point to relevant contributions both in the field of care and management as well as in healthcare training processes. The study highlights the importance of strengthening nursing’s presence in highly complex and high-risk scenarios, such as those experienced in tactical operations. It also highlights the urgent need to invest in technologies adapted to the realities of the field, supporting rapid, safe, and effective emergency response. At the same time, the teaching-learning axis also stands out as a strategic dimension, especially when considering the importance of continuing education in tactical first aid, with an emphasis on realistic practice and internationally recognized response protocols.

The need to expand spaces for listening and valuing military nursing within and outside of its institutions of origin is also reinforced, contributing to the recognition of this field as strategic in public health. Thus, we recommend further research to help understand the meanings attributed to care in operational contexts, as well as the ethical, managerial, and training challenges involved in this practice.

## FINAL CONSIDERATIONS

This study highlighted the strategic role of military nursing in TPHC, highlighting its leading role in staff training, resource management, and overcoming structural and communication barriers in high-risk settings. The results demonstrated that these professionals not only execute operational protocols but also play an active role in transforming institutional practices, acting as trainers, managers, and coordinating agents in environments characterized by tension, scarcity, and high complexity. Through the lens of Giddens’ Structuration Theory, it was possible to understand how military nurses produce and reproduce practices that shape the healthcare field and contribute to strengthening operational health.

Qualitative analysis revealed that, even faced with rigid hierarchies and material limitations, military nursing professionals demonstrate a strong commitment to improving care, investing in training, promoting logistical innovations, and adapting practices to changing realities. This agency reveals a dynamic field of activity, still largely overlooked in Brazilian scientific literature, that deserves recognition and institutional strengthening. Despite this, the scarcity of technical and scientific publications by authors affiliated with the armed forces-often more focused on service and leadership than on academic production-has limited the dissemination of these experiences, hindering the construction of knowledge networks and the advancement of evidence in the field of military nursing.

Given this scenario, the urgency of greater investment in ethical and legal frameworks and institutional guidelines that promote the integration of teaching, research, and practice in TPHC is reinforced. Strategies must be created to increase the visibility of military nursing in academic circles, encourage scientific production on the topic, and ensure infrastructure and ongoing training for these professionals. In times of crisis, such as wars, urban violence, pandemics, and complex emergencies, the coordinated action of the Armed Forces with public health systems depends, to a large extent, on the strengthening of areas such as TPHC, and military nursing, in this context, proves to be a fundamental pillar for an effective, humane, and evidence-based response.

## Data Availability

The research data are available only upon request.
