# Supplementary material for: Military nursing in tactical pre-hospital care: structure, action, and challenges in high-risk contexts
Source: Rev Bras Enferm. 2026 Jan 26;79:e20250183. doi: 10.1590/0034-7167-2025-0183 (PMC12844542; doi:10.1590/0034-7167-2025-0183)
Supplement: Translation

# Translation: A enfermagem militar no atendimento pré-hospitalar tático: estrutura, ação e desafios em contextos de risco

**DOI:** 10.1590/0034-7167-2025-0183pt

**Authors:** Fernanda Idamares da Silva Souza, Priscilla Valladares Broca, Fábio José de Almeida Guilherme, Débora Fernanda Haberland, Thiago de Souza Louro, Eric Rosa Pereira, Thiago Augusto Soares Monteiro da Silva, Alexandre Barbosa de Oliveira

**Affiliations:** I Universidade Federal do Rio de Janeiro. Rio de Janeiro, Rio de Janeiro, Brasil; II Força Aérea Brasileira. Rio de Janeiro, Rio de Janeiro, Brasil; III Universidade Federal do Estado do Rio de Janeiro. Rio de Janeiro, Rio de Janeiro, Brasil; IV Exército Brasileiro. Manaus, Amazonas, Brasil; V Faculdade Souza Marques. Rio de Janeiro, Rio de Janeiro, Brasil

**Keywords:** Enfermagem Militar, Papel do Profissional de Enfermagem, Servicos Médicos de Emergência, Desastres, Guerras e Conflitos Armados.

## Abstract

**Objetivos:**

compreender, à luz da Teoria da Estruturação de Anthony Giddens, como profissionais de enfermagem militar atuam no atendimento pré-hospitalar tático, evidenciando práticas formativas, gerenciais e assistenciais em contextos de risco, e desafios estruturais que tensionam sua prática.

**Métodos:**

estudo qualitativo, descritivo, com 15 participantes recrutados por amostragem em cadeia. Coleta ocorreu entre março/2023 e janeiro/2024 utilizando entrevistas semiestruturadas. O *corpus* foi processado no *Interface de R pour les Analyses Multidimensionnelles de Textes et de Questionnaires* e analisado por Classificação Hierárquica Descendente.

**Resultados:**

emergiram quatro classes, organizadas em três eixos: protagonismo na formação; gestão de recursos em ambientes hostis; desafios relacionados à comunicação e escassez de insumos.

**Conclusões:**

a enfermagem militar demonstra agência ao adaptar práticas e produzir inovação mesmo diante de estruturas hierárquicas rígidas. Há necessidade de institucionalização do atendimento pré-hospitalar tático, investindo em formação, tecnologias e produção científica que consolidem esse campo como estratégico para a saúde operacional.

## INTRODUÇÃO

A atuação da enfermagem militar em cenários de guerra e de confrontos armados tem sido historicamente registrada, mas nota-se uma lacuna na produção científica que examine, de maneira sistemática, a atuação da enfermagem no âmbito do atendimento pré-hospitalar tático (APH-T), sobretudo no cenário brasileiro. A literatura disponível concentra-se majoritariamente em relatos históricos ou análises conceituais, o que evidencia uma escassez de estudos empíricos que investiguem os desafios operacionais, formativos e gerenciais enfrentados por esses profissionais em contextos de risco^(1-3)^.

O APH-T surge, assim, como uma resposta às exigências impostas por contextos de guerra e conflitos armados, configurando-se como modelo de cuidado emergencial em saúde voltado a situações de elevado risco. Para tanto, foram desenvolvidos protocolos adaptados a diferentes realidades táticas - civis e militares -, com vistas a qualificar a assistência prestada e aprimorar a resposta imediata em campo. Um exemplo emblemático dessa sistematização é o *Tactical Combat Casualty Care* (TCCC), instituído em 1996 pelo Comando Especial de Guerra Naval e pelo Comando de Operações Especiais dos Estados Unidos da América (EUA), com o objetivo central de reduzir as baixas em combate, assegurar o atendimento adequado aos feridos e contribuir para o sucesso das missões operacionais^(4)^.

O TCCC é fundamentado em três princípios básicos: *Care Under Fire* (Cuidado sob Fogo); *Tactical Field Care* (Cuidado em Campo Tático); e *Casualty Evacuation Care* (Cuidados na Evacuação Tática). Esses princípios habilitam o profissional de saúde militar, seja ele enfermeiro ou não, a agir de maneira eficaz, mesmo sob fogo inimigo, oferecendo cuidados emergenciais de suporte básico e avançado de vida durante o atendimento e na evacuação segura dos feridos no campo tático^(4)^.

Os profissionais envolvidos no APH-T são classificados em três níveis de atuação, conforme especificado pelo *Department of Defense Health Agency* (DHA) *Joint Trauma System* (JTS), a saber: nível I - todos os membros do serviço militar; nível II - militares não médicos em operações de combate; e nível III - profissionais médicos militares, paramédicos, enfermeiros e soldados. Essa classificação é amplamente adotada de forma internacional, sendo adaptada por Forças Armadas e agências de segurança pública em diversas partes do mundo^(5-7)^. Nesse contexto, a enfermagem tem se consolidado como uma profissão central na promoção e desenvolvimento das capacitações. No Brasil, enfermeiros e técnicos de enfermagem vêm atuando como instrutores e agentes educadores de APH-T^(8)^.

Quando a crise sanitáriada pandemia de COVID-19 atingiu os EUA, profissionais de saúde militar, incluindo enfermeiros, médicos e técnicos, foram mobilizados para reforçar as equipes nos hospitais civis. Em 2020, uma força-tarefa composta por 140 militares da saúde viabilizou, em tempo recorde, a distribuição de vacinas, diagnósticos e terapias seguras e eficazes, contribuindo diretamente para a preservação de mais de um milhão de vidas^(3)^. Tais evidências reforçam a relevância estratégica da enfermagem militar no fortalecimento da saúde operacional tanto no âmbito das Forças Armadas quanto na interface com a saúde pública civil.

À luz da Teoria da Estruturação de Anthony Giddens^(9)^, compreende-se que a atuação da enfermagem militar no APH-T se desenvolve em um campo tensionado pela dualidade entre estrutura e agência. De um lado, normas institucionais, códigos hierárquicos e protocolos operacionais conformam um conjunto de regras e recursos que limitam e regulam a prática profissional; de outro, os enfermeiros militares exercem agência (protagonismo) ao reinterpretar, negociar e ressignificar essas estruturas em sua atuação cotidiana. Assim, o APH-T no campo militar não deve ser compreendido apenas como um *locus* de aplicação técnica de normas preestabelecidas, mas como um espaço social dinâmico e relacional em que as práticas são continuamente (re)construídas em interação com as condições estruturais. Essa perspectiva permite reconhecer que os desafios enfrentados pela enfermagem militar não se limitam a barreiras técnicas ou logísticas, mas dizem respeito a processos estruturantes mais amplos, com implicações diretas para a consolidação de práticas baseadas em evidências e para o fortalecimento da enfermagem militar enquanto campo de saber e atuação especializada^(1,8)^.

Diante desse cenário, torna-se fundamental analisar de que maneira os constrangimentos estruturais e operacionais interferem na prática dos profissionais de enfermagem militar no APH-T, bem como compreender os efeitos dessas tensões na produção de conhecimento, na qualificação da assistência prestada e na institucionalização da enfermagem militar como uma dimensão estratégica da saúde operacional.

## OBJETIVOS

Compreender, à luz da Teoria da Estruturação de Anthony Giddens, como profissionais de enfermagem militar atuam no APH-T, evidenciando práticas formativas, gerenciais e assistenciais em contextos de risco, e os desafios estruturais que tensionam sua prática.

## MÉTODOS

### Aspectos éticos

O protocolo de pesquisa foi submetido e aprovado pelo Comitê de Ética em Pesquisa da instituição proponente. Todos os participantes assinaram o Termo de Consentimento Livre e Esclarecido. Para assegurar o anonimato, os entrevistados foram identificados por códigos alfanuméricos, sendo utilizado o prefixo “PE”, para profissionais de enfermagem, seguido de um número sequencial correspondente à ordem das entrevistas.

### Tipo de estudo

Trata-se de pesquisa descritiva e exploratória, de abordagem qualitativa, desenvolvida conforme as diretrizes metodológicas recomendadas pelo *COnsolidated criteria for REporting Qualitative research*.

### Procedimentos metodológicos

A coleta de dados foi realizada entre março de 2023 e janeiro de 2024. O recrutamento dos participantes ocorreu por meio da técnica de *snowball sampling* (amostragem em cadeia ou “bola de neve”), amplamente reconhecida na pesquisa qualitativa como uma estratégia eficaz para acessar grupos específicos ou de difícil localização, especialmente em contextos institucionais restritos como o meio militar^(10)^. O primeiro informante, um enfermeiro das Forças Armadas brasileiras, foi identificado durante um evento promovido por uma liga acadêmica de enfermagem em urgência e emergência da Universidade Federal do Rio de Janeiro, ocasião em que a pesquisadora principal atuava como presidente da organização estudantil. A partir desse contato inicial, os demais participantes foram progressivamente indicados pelos próprios entrevistados. Esse processo em rede permitiu o acesso a profissionais com *expertise* no APH-T, favorecendo a diversidade de experiências e perfis até o alcance da saturação teórica dos dados.

### Cenário do estudo

As entrevistas foram realizadas individualmente, de forma remota, por meio da plataforma *Google Meet^®^*. Essa modalidade virtual de coleta foi adotada por oferecer maior flexibilidade aos participantes, considerando que muitos deles se encontravam em serviço ativo ou mobilizados em diferentes regiões do território nacional. Além das barreiras geográficas, a estratégia também viabilizou a superação de barreiras logísticas.

### Composição da amostra e critérios de seleção

A amostra deste estudo foi composta por 15 profissionais militares, entre enfermeiros e técnicos de enfermagem, vinculados às Forças Armadas (Marinha, Exército ou Aeronáutica) e às Forças Auxiliares, como as Polícias Militares e os Corpos de Bombeiros Militares estaduais. Inicialmente, foram recrutados 16 participantes; no entanto, um deles foi excluído da etapa de entrevista devido à sua designação para missão operacional durante o período de coleta, totalizando 15 participantes ativos na produção de dados.

Foram incluídos profissionais com vínculo institucional com as Forças Armadas ou forças de segurança pública, formação técnica ou superior em enfermagem e experiência mínima de um ano de atuação direta no APH-T, a fim de assegurar a participação de sujeitos com vivência no campo operativo investigado. Foram excluídos profissionais afastados de suas funções no momento da coleta de dados, seja por motivo de licença ou por pertencerem à reserva (remunerada ou não remunerada), garantindo, assim, a atualidade e a pertinência dos relatos colhidos.

### Coleta e organização de dados

A coleta de dados foi realizada por meio de entrevistas semiestruturadas, organizadas em dois eixos centrais. O primeiro eixo teve como foco a caracterização sociodemográfica e profissional dos participantes, contemplando variáveis como sexo, faixa etária, tempo de formação na enfermagem (nível técnico e/ou superior) e tempo de atuação no APH-T. O segundo eixo abrangeu questões abertas, voltadas à compreensão da experiência profissional no contexto do APH-T, incluindo temas como atribuições da enfermagem militar, desafios enfrentados no campo operacional, processos de formação e capacitação contínua, barreiras logísticas, e percepções sobre a comunicação interprofissional em ambientes táticos.

As entrevistas foram gravadas em áudio. Em seguida, foram transcritas na íntegra e submetidas à validação pelos próprios entrevistados, com o objetivo de assegurar a fidedignidade e a credibilidade das informações coletadas. A duração média das entrevistas foi de 150 minutos, variando entre 60 e 150 minutos.

A coleta foi encerrada com base no critério da saturação teórica, ou seja, quando as entrevistas passaram a apresentar recorrência temática e ausência de dados novos relevantes, conforme preconizado na literatura sobre a abordagem qualitativa^(11,12)^.

### Análise e interpretação dos dados

A análise dos dados foi conduzida por meio da Classificação Hierárquica Descendente (CHD), utilizando o *software Interface de R pour les Analyses Multidimensionnelles de Textes et de Questionnaires*, versão 0.7 alpha 2. Este *software* é amplamente reconhecido por sua robustez na análise de dados textuais e na extração de padrões lexicais em pesquisas qualitativas.

O processo analítico envolveu as seguintes etapas: 1) pré-processamento do *corpus* textual, com padronização da ortografia, eliminação de palavras de ligação sem carga semântica e preparação do material para segmentação; 2) segmentação do *corpus* em Unidades de Contexto Inicial e Unidades de Contexto Elementar, permitindo a análise detalhada das frequências e relações entre os termos mais significativos; 3) processamento da CHD, que organizou os dados em um dendrograma de classes lexicais, identificando padrões linguísticos e semânticos recorrentes nos discursos dos participantes; e 4) interpretação das classes, considerando os contextos e relações estabelecidas entre os discursos dos participantes à luz da Teoria da Estruturação de Giddens, que fundamentou a discussão dos achados.

A interpretação das classes foi guiada pelos fundamentos da Teoria da Estruturação de Anthony Giddens^(9)^, com ênfase nos conceitos de: “estrutura”, entendida como o conjunto de normas, regras e recursos institucionalizados que organizam e condicionam a ação; “agência”, entendida como a capacidade dos sujeitos de agir reflexivamente, resistir, adaptar ou transformar essas estruturas; e “rotina”, compreendida como o modo pelo qual práticas são reproduzidas ou modificadas no cotidiano. Essa abordagem permitiu captar as dinâmicas relacionais entre as dimensões estruturais e as ações dos profissionais de enfermagem militar no contexto do APH-T.

## RESULTADOS

### Perfil sociodemográfico e profissional

A amostra foi composta por 15 profissionais de enfermagem militar, dos quais 13 (86,7%) eram do sexo masculino e dois (13,3%) do sexo feminino. Em relação à formação profissional, 53% dos participantes possuíam graduação em enfermagem e 47% eram técnicos de enfermagem, evidenciando uma composição diversificada quanto ao nível de escolaridade e atribuições no campo assistencial.

A análise da faixa etária revelou um grupo profissional experiente, com 100% dos entrevistados situados na faixa etária acima de 36 anos. O tempo de formação variou entre cinco e 20 anos, tanto para os profissionais de nível técnico quanto para os profissionais de nível superior. Já a experiência específica no APH-T, considerando a atuação nas Forças Armadas e Forças Auxiliares, oscilou entre 5 e 15 anos, o que reforça a *expertise* e a maturidade prática dos sujeitos envolvidos na pesquisa.

### Análise lexical do *corpus* textual

A análise lexical do *corpus* revelou 715 segmentos de texto, dos quais 586 foram aproveitados, representando 81,96% do material analisado. O total de ocorrências foi de 25.105 palavras, formas ou vocábulos, sendo 2.300 palavras distintas e 2.001 palavras com uma única ocorrência (hápax).

A CHD possibilitou a organização do *corpus* em quatro classes distintas, resultando na divisão dos discursos em duas ramificações principais. A primeira ramificação foi composta exclusivamente pela classe 2, enquanto a segunda ramificação agregou as classes 1, 3 e 4. O dendrograma gerado evidencia a segmentação dos discursos e a coesão semântica dos conteúdos analisados (Figura 1).

**Figura 1:** Dendrograma da Classificação Hierárquica Descendente, Rio de Janeiro, Rio de Janeiro, Brasil

*Fonte: software IRaMuTeQ® 0.7 alpha 2.*

A análise semântica e categorial permitiu a identificação de três grandes temas centrais, organizados a partir das quatro classes léxicas. Essa estrutura possibilitou uma compreensão aprofundada das dinâmicas de atuação da enfermagem militar no APH-T, evidenciando desafios, funções estratégicas e barreiras operacionais enfrentadas no contexto tático.

### Interpretação das classes emergentes

A análise lexical do *corpus* revelou quatro classes léxicas, organizadas em três grandes temas centrais que expressam diferentes dimensões da atuação da enfermagem militar no contexto do APH-T. Essa perspectiva permite compreender que as práticas da enfermagem militar não se reduzem à aplicação mecânica de protocolos pré-estabelecidos. Pelo contrário, envolvem processos contínuos de produção e reprodução social, nos quais rotinas operacionais são recriadas reflexivamente, articulando conhecimento tácito, improviso técnico e adequação situacional.

Cada uma das classes identificadas, acompanhada dos segmentos de texto correspondentes, derivados diretamente das falas dos participantes, é apresentada a seguir com o propósito de ilustrar as dimensões empíricas que fundamentam os temas emergentes.

### Reproduzindo e transformando a prática: profissionais de enfermagem militar como agentes de formação (classe 2)

Esta categoria evidencia o papel estruturante dos profissionais de enfermagem militar como agentes formadores capazes de reproduzir e transformar práticas institucionais no contexto do APH-T. A formação no APH-T ocorre em múltiplos níveis e cenários, sendo atravessada por hierarquias, tradições e lacunas formativas que exigem do profissional de enfermagem um protagonismo contínuo na qualificação das equipes, sobretudo nos níveis 2 e 3 de atuação. Nesse processo, os enfermeiros e técnicos não apenas reproduzem as normas institucionais, mas também contribuem para sua ressignificação, ao adequar os conteúdos à realidade operacional, promovendo adaptações metodológicas e disputando espaços de legitimidade técnica e política.

*O enfermeiro atua fortemente na formação, principalmente dos militares dos níveis 2 e 3, que são os que vão estar na linha de frente*. [...] *no Brasil, poucos médicos e enfermeiros atuam nessa área. A gente precisa garantir como esse conhecimento será transmitido, praticado e consolidado no nível 2 e no nível 3.* (PE1)

*Na minha trajetória, sempre me vi ensinando o tempo todo. Quando um enfermeiro recém-chegado do curso de oficiais assume a função, ele geralmente tem pouca ou nenhuma experiência no contexto tático* [...]*. Cabe a mim começar do zero, treiná-lo. Isso vale para médicos, técnicos de enfermagem, enfim, para todos.* (PE10)

*Dentro do exército, a gente vê o profissional de saúde como multiplicador do atendimento pré-hospitalar tático. É ele que vai estar nos confins do Brasil, capacitando e reciclando o combatente.* (PE9)

*Eu briguei muito para equiparar o enfermeiro com o médico para o atendimento pré-hospitalar tático avançado, e eu consegui. Estamos lá no nível avançado - médicos e enfermeiros. Se eu não estivesse lá representando a categoria, se tivesse ido um médico, por exemplo, a legislação seria outra.* (PE6)

### Gestão e estruturação de ações em ambientes hostis: estratégias da enfermagem militar no atendimento pré-hospitalar tático (classe 4)

Esta categoria evidencia a atuação estratégica da enfermagem militar como agente organizador e articulador das ações logísticas e assistenciais no contexto do APH-T. Em situações marcadas por escassez de recursos, exigência técnica e riscos iminentes, os profissionais de enfermagem desempenham papel central não apenas na assistência direta, mas também na gestão de insumos, na coordenação de equipes e no planejamento das respostas táticas.

Os trechos a seguir ilustram os principais eixos dessa classe:

*Se enfrentamos um desastre, precisamos calcular: quantos torniquetes podem ser utilizados? Quantas bandagens serão necessárias? Esse tipo de discussão é essencial para garantir a assistência.* (PE9)

*Quando capacito o militar no atendimento tático, estou também lidando com questões gerenciais. Esse assessoramento do especialista torna a resposta mais eficaz, principalmente em desastres. É preciso planejar o que será difundido à tropa, quais melhorias implementar. Trabalho com gerenciamento, ensino e logística.* (PE9)

*O trabalho militar coloca o enfermeiro e o técnico em situações de extremo desafio, exigindo raciocínio clínico, liderança e aplicação de conhecimentos em ambientes hostis e sob alto estresse.* (PE11)

*As próprias instituições de segurança pública ainda não compreenderam que o modelo de ‘cena segura’ é idealizado para países sem histórico de guerra. No Brasil, especialmente no Rio de Janeiro, vivemos em permanente conflito urbano.* (PE1)

### Barreiras estruturais e desafios tecnológicos no atendimento pré-hospitalar tático: comunicação e recursos escassos (classes 1 e 3)

Esta categoria articula dois eixos de análise centrais à realidade da enfermagem militar no APH-T: a comunicação interprofissional (classe 1) e a escassez de recursos materiais e tecnológicos (classe 3).

No que tange à classe 1, a comunicação em contextos táticos é tensionada por estruturas hierárquicas inflexíveis, comprometendo a articulação entre os membros da equipe multiprofissional. A dificuldade de reconhecimento da autoridade técnica do enfermeiro e o desconhecimento de protocolos específicos por parte de outros profissionais geram ruídos, atrasos e até falhas na assistência.

*Por conta da doutrina militar, às vezes tentamos discutir um procedimento com o médico assistencial e mencionamos o TC3 ou outro protocolo específico, e isso complica, pois ele não entende do que estamos falando. A situação se agrava quando lidamos com oficiais que não estão abertos ao aprendizado. Já tive muitos problemas chegando em emergência com um paciente acidentado por causa disso.* (PE11)

*A hierarquia atrapalha, sim. Muitas vezes, quem está na posição de comando não é da saúde e não entende certas recomendações técnicas. Antes da crise, há resistência; depois que a crise se instala, todos se calam e você vira o “dono da verdade”. Já perdi paciente por isso. É difícil, pois, mesmo com orientação prévia, nem sempre escutam. A autoridade hierárquica se impõe e temos que cumprir, mesmo quando não é a melhor decisão*. (PE15)

*A comunicação dentro da própria tropa é, muitas vezes, o problema principal. Em algumas operações, conseguimos entrar na comunidade com a patrulha, deixamos a ambulância fora e seguimos com as mochilas e equipamentos. Porém, por falha de comunicação, os pontos estratégicos de entrada e evacuação nem sempre são definidos de forma eficaz, o que compromete toda a operação.* (PE2)

*A comunicação, em alguns momentos, é falha. Precisamos construir confiança para superar essas dificuldades. Hoje em dia, mesmo com os celulares, ainda existem ruídos que impactam diretamente o atendimento.* (PE6)

Já na classe 3, são contemplados desafios enfrentados no APH-T relacionados à precariedade de insumos essenciais, como torniquetes, agentes hemostáticos e bandagens especializadas, o que impõe barreiras significativas à prática assistencial. Mesmo com protocolos consolidados, a ausência de materiais compromete sua aplicação e efetividade.

*Não adianta treinar e treinar se, na hora da ocorrência, não houver material de qualidade. Por exemplo, se uma pessoa é baleada e usamos um torniquete, esse item é descartável. Depois, precisa ser reposto. E isso custa caro.* (PE7)

*Hoje, se quisermos material para trabalhar, temos que comprar. Muitos profissionais já se conscientizaram disso: se não comprar, pode morrer. Por isso, é comum ver torniquetes pessoais presos às fardas.* (PE2)

*Muitos dos materiais que utilizamos no atendimento pré-hospitalar tático chegam por meio de intercâmbios internacionais, especialmente com os norte-americanos. Eles nos doam. No entanto, quando esses itens vencem e não há um novo intercâmbio programado, somos obrigados a comprá-los do próprio bolso.* (PE11)

## DISCUSSÃO

Os resultados indicaram a predominância de profissionais do sexo masculino no APH-T, o que reflete a herança institucional das Forças Armadas, marcadas por uma cultura organizacional que privilegia força física, disciplina rígida e operacionalidade tática^(13,14)^. Todos os entrevistados tinham mais de 36 anos, o que sinaliza um corpo profissional experiente, com trajetória consolidada em contextos de risco elevado.

Observou-se ainda que os profissionais entrevistados buscam, de forma ativa, impulsionar a sua preparação para atuação em cenários complexos - que envolvem confrontos armados, violência urbana e desastres^(15)^. Essa tendência também é relatada em estudos realizados em zonas de conflito na Ucrânia e em missões da Organização do Tratado do Atlântico Norte, onde enfermeiros militares acumulam atribuições logísticas, formativas e clínicas, sendo reconhecidos como líderes em campo^(16-18)^.

À luz da Teoria da Estruturação de Anthony Giddens^(9)^, é possível interpretar que as classes expressam um processo dinâmico em que os sujeitos, ao mesmo tempo em que são condicionados pelas estruturas organizacionais e normativas do campo militar, também exercem agência, isto é, a capacidade de agir, adaptar e transformar essas mesmas estruturas.

A classe 2, que evidenciou a atuação do profissional de enfermagem como agente formador, reafirma os pressupostos da Teoria da Estruturação de Giddens^(9)^, pois revela como esses sujeitos reconfiguram normas institucionais a partir de sua prática cotidiana. Ao mediar saberes táticos com a técnica assistencial, os enfermeiros militares exercem “agência”, construindo soluções que são, ao mesmo tempo, aderentes ao contexto operacional e inovadoras do ponto de vista pedagógico^(4,8,15,19,20)^. Essa realidade é convergente com modelos de formação militar adotados por países como Israel e Reino Unido, onde enfermeiros têm papel central na educação para trauma tático, inclusive em missões civis^(21,22)^.

À luz da Teoria da Estruturação de Giddens^(9)^, a prática pedagógica exercida por esses profissionais expressa um processo dinâmico de agência, no qual a transmissão de saberes não se limita aos protocolos formais, mas envolve também a incorporação de conhecimentos tácitos adaptados às exigências do cotidiano militar.

A classe 4 expôs a capacidade da enfermagem militar de exercer funções gerenciais em ambientes marcados por escassez e imprevisibilidade. As práticas relatadas, como planejamento de treinamentos, gestão de insumos e coordenação de resposta tática, refletem a capacidade desses sujeitos de moldar a estrutura organizacional a partir da prática^(8,21)^. Essa agência, nos termos de Giddens, torna-se essencial em zonas instáveis, como demonstram as experiências recentes do Exército dos EUA e das Forças Armadas alemãs em apoio à população durante enchentes^(23,24)^.

À luz da Teoria da Estruturação de Giddens^(9)^, essa categoria reflete a relação dialética entre estrutura e ação: embora inseridos em um sistema normativo rígido, os enfermeiros militares exercem agência ao negociar, adaptar e transformar as práticas operacionais. Tal agência se manifesta na forma como esses profissionais gerenciam fluxos decisórios, otimizam a logística em campo e influenciam os processos de formação técnica e liderança em cenários críticos.

As barreiras à comunicação interprofissional (classe 1), por sua vez, revelam tensões entre a autoridade técnica da enfermagem e as normas hierárquicas da estrutura militar. A interoperabilidade entre profissionais de saúde e do comando operacional, apontada como desafio, está igualmente presente em outras realidades de resposta a emergências. A falta de compreensão mútua entre áreas técnicas e operacionais compromete a fluidez da assistência, demandando treinamentos interagências e protocolos de comunicação tática unificada^(13,25,26)^.

A classe 3 destacou a precariedade de insumos como barreira crítica à efetivação de protocolos internacionais de APH-T, como o TCCC. A escassez de torniquetes, agentes hemostáticos e equipamentos de proteção repercute diretamente na capacidade de resposta e na segurança das equipes. Nos EUA, legislações recentes vêm priorizando o financiamento de *kits* de trauma tático para unidades civis e militares, reconhecendo o papel dos enfermeiros táticos como provedores centrais do cuidado^(2-4,8,22)^. No Brasil, no entanto, a dependência de doações ou aquisição pessoal por parte dos profissionais evidencia a ausência de políticas estruturantes para a saúde operacional.

À luz da Teoria da Estruturação de Giddens^(9)^, compreende-se que, mesmo condicionados por estruturas rígidas, como normas militares, hierarquias institucionais e limitações orçamentárias, os profissionais de enfermagem exercem agência ao desenvolver estratégias para manter a eficácia assistencial em cenários de alto risco.

Essas evidências reforçam que, mesmo diante de constrangimentos estruturais, os profissionais de enfermagem militar exercem agência criativa, reinterpretando normas e adaptando práticas em defesa da vida. O referencial de Giddens permite compreender que a estrutura não é imutável, mas constantemente renegociada pelos sujeitos que a habitam.

Diante desse cenário, torna-se urgente incorporar experiências internacionais de sucesso, ampliar a institucionalização do APH-T como campo estratégico e investir na formação técnico-gerencial da enfermagem. O fortalecimento de redes colaborativas, a regulamentação normativa específica e a produção de evidências científicas robustas são caminhos promissores para consolidar a atuação da enfermagem militar no cenário contemporâneo de crises complexas.

### Limitações do estudo

Este estudo enfrentou limitações relacionadas à natureza específica do campo militar. Muitos profissionais de enfermagem militar estavam frequentemente em missões ou deslocamentos estratégicos quando foram contatados, o que dificultou o acesso a um número maior de participantes. Esse contexto, próprio da dinâmica institucional e cultural das corporações militares, acabou restringindo a possibilidade de ampliar a diversidade de vozes escutadas ao longo da pesquisa. Para estudos futuros, a articulação com setores institucionais poderá abrir caminhos para uma aproximação mais estruturada com diferentes unidades operacionais, ampliando o escopo e a representatividade dos achados.

### Contribuições para a área da enfermagem e da saúde

Os resultados desta investigação apontam para contribuições relevantes tanto no campo da assistência e gestão quanto nos processos de formação em saúde. O estudo evidencia a importância de fortalecer a presença da enfermagem em cenários de alta complexidade e risco, como aqueles vivenciados em operações táticas. Torna-se visível a urgência de investir em tecnologias adaptadas à realidade do campo que apoiem uma atuação rápida, segura e eficaz nos atendimentos emergenciais. Em paralelo, o eixo do ensino-aprendizagem também se destaca como uma dimensão estratégica, especialmente quando se considera a importância da formação continuada em primeiros socorros táticos, com ênfase na prática realística e nos protocolos de resposta internacionalmente reconhecidos.

Reforça-se ainda a necessidade de ampliar os espaços de escuta e valorização da enfermagem militar dentro e fora das instituições de origem, contribuindo para o reconhecimento desse campo como estratégico na saúde coletiva. Nesse sentido, recomenda-se o aprofundamento de investigações que ajudem a compreender os sentidos atribuídos ao cuidado em contextos operacionais, bem como os desafios éticos, gerenciais e formativos que envolvem essa prática.

## CONSIDERAÇÕES FINAIS

Este estudo evidenciou a atuação estratégica da enfermagem militar no APH-T, destacando seu protagonismo na formação de pessoal, na gestão de recursos e na superação de barreiras estruturais e comunicacionais em contextos de risco elevado. Os resultados demonstraram que esses profissionais não apenas executam protocolos operacionais, mas também exercem papel ativo na transformação das práticas institucionais, atuando como formadores, gestores e agentes articuladores em ambientes marcados por tensão, escassez e alta complexidade. A partir das lentes da Teoria da Estruturação de Giddens, foi possível compreender como os enfermeiros militares produzem e reproduzem práticas que moldam o campo assistencial e contribuem para o fortalecimento da saúde operacional.

A análise qualitativa revelou que, mesmo diante de rígidas hierarquias e limitações materiais, os profissionais de enfermagem militar demonstram elevado compromisso com a qualificação do cuidado, investindo em capacitações, promovendo inovações logísticas e adaptando práticas conforme a realidade do terreno. Essa agência revela um campo de atuação dinâmico, ainda pouco visibilizado na literatura científica nacional, que merece reconhecimento e fortalecimento institucional. Apesar disso, a escassez de publicações técnico-científicas por autores vinculados às Forças Armadas - frequentemente mais voltadas ao serviço e à liderança do que à produção acadêmica - tem limitado a difusão dessas experiências, dificultando a construção de redes de conhecimento e o avanço das evidências no campo da enfermagem militar.

Diante desse cenário, reforça-se a urgência de maior investimento em bases ético-legais e diretrizes institucionais que promovam a integração entre ensino, pesquisa e prática no APH-T. É necessário criar estratégias que ampliem a visibilidade da enfermagem militar nos espaços acadêmicos, incentivem a produção científica sobre o tema, e assegurem infraestrutura e formação contínua para esses profissionais. Em tempos de crise, como guerras, situações de violência urbana, pandemias e emergências complexas, a atuação articulada das Forças Armadas com os sistemas de saúde pública depende, em grande medida, do fortalecimento de áreas como o APH-T, e a enfermagem militar, neste contexto, mostra-se um pilar fundamental para a resposta eficaz, humanizada e baseada em evidências.

## Data Availability

Os dados de pesquisa só estão disponíveis mediante solicitação.
